# Comparison between Different Groups of Vegetarianism and Its Associations with Body Composition: A Literature Review from 2015 to 2021

**DOI:** 10.3390/nu14091853

**Published:** 2022-04-28

**Authors:** Tatiana Fontes, Luis Monteiro Rodrigues, Cíntia Ferreira-Pêgo

**Affiliations:** CBIOS—Universidade Lusófona’s Research Center for Biosciences and Health Technologies, Av. Campo Grande 376, 1749-024 Lisbon, Portugal; f6776@ulusofona.pt (T.F.); monteiro.rodrigues@ulusofona.pt (L.M.R.)

**Keywords:** vegetarian diet, vegan diet, body composition, body mass index, literature review

## Abstract

Background: Vegetarian and vegan diets have become increasingly popular in the last years for many reasons, including their association with various health benefits when compared to omnivorous diets. The main objective of the study was to collect recent (2015–2021) scientific evidence for potential implications between a vegetarian/vegan diet and an individuals’ body composition. Methods: A literature search was conducted in PubMed, with 22 studies selected for inclusion in our collective evaluation. Of the 22 studies included, there were 12 randomized controlled trials, 1 nonrandomized controlled trial, 1 comparative study, and 8 cross-sectional. The overall sample included in this study consists of 436,178 participants, 10,090 of whom were vegetarians, 5044 vegans, and 421,044 omnivores. Results: Most studies, 17 out of 22, reported a significant positive relationship between a plant-based diet and body composition. Conclusion: There is scientifically sound evidence that vegan or vegetarian diets are associated with weight and body mass index reduction and, in some cases, fat mass distribution changes.

## 1. Introduction

A vegetarian diet predominantly consists of foodstuffs of plant origin (cereals, vegetables, root crops, oilseeds, fruit, and nuts) and tends to exclude any type of meat, fish, or animal product or subproduct [1,2]. The vegetarian diet can be further divided into several subgroups: ovo-vegetarian (includes eggs), ovo-lacto-vegetarian (includes eggs and dairy products), and lacto-vegetarian (includes dairy products) [1,2]. Flexitarian diets are another type of vegetarianism, although they are less stringent regarding the consumption of meat and fish [3,4]. The vegan diet is the strictest, as no animal-derived foods (including honey) are consumed [1]. Individuals who follow a vegan diet are normally concerned not only with what they eat but also with the environment and animal protection issues [5]. Vegetarian diets have become increasingly popular, in part because they have been associated with many health benefits when compared to omnivorous diets [5]. Foods in these diets are typically rich in fiber, phytoestrogens, antioxidants, phytochemicals, n-3 fatty acids, and poor in cholesterol and saturated fat, allegedly contributing to a reduction in risk of cardiovascular disease, diabetes, and obesity, among others [2,5,6]. According to the World Health Organization (WHO), about 1.2 billion people in the world were overweight at the turn of the 20th century [2,7]. It is now known that obesity is a complex disease, and its comorbidities are responsible for thousands of deaths worldwide. It is therefore extremely important to find more effective solutions to combat this pandemic [2,8,9]. Recent reports support the claim that a well-balanced vegetarian diet may be associated with a lower body mass index (BMI) compared to other diets [2,10]. These diets have also been linked to a wide range of beneficial nutritional, metabolic, and health outcomes, including lipid metabolism, bone mineral density, body weight, body fat percentage, obesity-related meta-inflammatory profile, and diabetes risk [2,4,10]. Its therapeutical potential to address obesity has been suggested [2]. However, despite these associations, there is still much controversy related to this type of diet and its true efficiency and safety [2,5]. Low levels of vitamin B12 and the increase in serum homocysteine are frequently raised concerns [2,5]. Thus, it has become increasingly important to understand the health benefits that a vegetarian diet might bring, especially in terms of weight loss and fighting obesity. This study aims to review the most recent scientific evidence regarding the potential associations and consequences relating to vegetarian/vegan diets and human body composition.

## 2. Materials and Methods

### 2.1. Search Strategy and Inclusion and Exclusion Criteria

According to the PRISMA statement 2020, we identified all potentially relevant articles through a computerized search in PubMed (from April 2021 to September 2021). Search terms included keywords “vegetarians”, “vegetarianism”, “vegetarian diet”, “vegan”, “veganism”, and “vegan diet” used in combination as MeSH terms and text words such as “body mass index” and “body composition”. All manuscripts that did not compare the vegetarian or vegan diet with another diet, studies not performed in humans, studies performed in children or adolescents, as well as review articles, or systematic reviews/meta-analyses were excluded from this review. The manuscripts’ inclusion decision was initially based on the title, then on the study abstract, and finally on the complete study manuscript. All studies with body composition-related values from a vegetarian/vegan population and not meeting any of the non-inclusion criteria were included, even if body composition measurements were not the main objective of the study.

### 2.2. Data Collection

**Figure 1:**
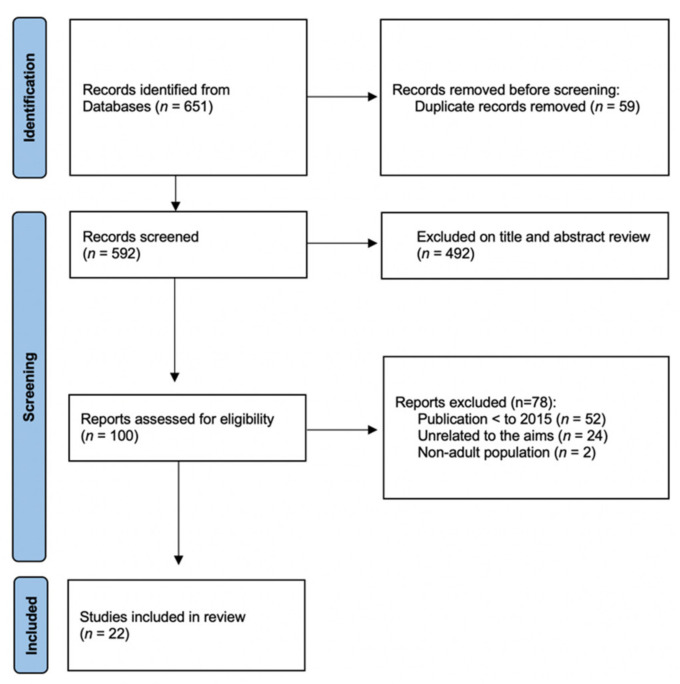
Flowchart for search strategy.

In the first identification phase, 651 articles were found, which was reduced to 592 studies after the exclusion of duplicates. In the second selection phase, 492 articles were excluded after reading the title and abstract, as they did not meet the purpose of the research. This resulted in 100 studies. In the third eligibility phase, from those 100 articles selected for thorough reading, 78 were excluded: 52 were excluded because their publication was prior to 2015, 24 were excluded because their research was not related to the objective of this study and, finally, 2 were excluded because the studied population was children/adolescents. In the fourth inclusion phase, 22 articles that met all the inclusion criteria and the research objective were selected for the present review after detailed evaluation by the authors (Figure 1). Of the 22 studies included, there were 12 randomized controlled trials, 1 nonrandomized controlled trial, 1 comparative study, and 8 cross-sectional. The overall sample included in this study consisted of 436,178 participants, 10,090 of whom were vegetarians, 5044 vegans, and 421,044 omnivores.

## 3. Results

**Figure 2:**
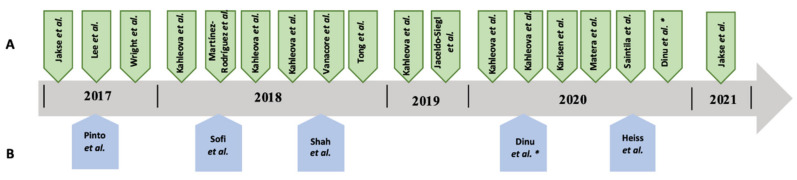
Epidemiological studies included in the review: (**A**) significant positive relationship, and (**B**) no demonstrated effects between vegetarian/vegan diet and body composition. *First intervention no demonstrated effects, between vegetarian/vegan diet and body composition but second interview found a significant positive relation.

In most studies, we found that sociodemographic characteristics were not statistically different. Study participant ages ranged from 18 to 75 years old. Most studies found a significant positive relationship between vegetarian/vegan diet and body composition, as shown in Figure 2.

### 3.1. Experimental Studies

Table 1 includes 12 randomized controlled trials and 1 nonrandomized controlled trial. The total sample consisted of 1388 participants, 650 of whom were vegans, 144 vegetarians, and 594 omnivores. At the beginning of each study, the participants in both groups (intervention and control) had identical weight and BMI, with the exception of the study by Jakse [11], where the intervention group started with a higher weight. The vast majority of studies observed a significant decrease in body weight and BMI [11,12,13,14,15,16,17,18,19] in the intervention groups (vegan or vegetarian diet) compared to the control groups (omnivorous diet). Likewise, some studies also found a significant reduction in visceral fat and fat mass only in the intervention group [11,14,16,17,18,20]. Martínez-Rodríguez et al. [21] verified that women who suffered from fibromyalgia and consumed an ovo-lacto-vegetarian diet had better results in reducing both pain and body composition, with an increase in muscle mass and a decrease in fat mass. On the other hand, the studies by Sofi et al. [22] and Shah et al. [23] did not observe differences in weight loss and BMI between the diet groups.

### 3.2. Descriptive Studies

Table 2 summarizes eight cross-sectional studies and one comparative study. The total sample consisted of 434,790 participants, 4394 of whom were vegans, 9946 vegetarians, and 420,450 omnivores. The majority of studies observed a significant decrease in body weight and BMI in individuals who consumed a vegetable-based diet [5,25,26,27,28,29]. The study by Tong et al. [30] found that white individuals who did not consume meat had lower values for body weight, BMI, waist circumference, and fat percentage. However, the same study found that these values were not as pronounced in the Indian population, although those who did not consume meat continued to present lower values for all parameters. Our research also found that some studies [31,32] did not observe statistically significant differences between diet groups, either for BMI or for other markers of body composition.

## 4. Discussion

This review aimed to collect recently published scientific evidence related to vegetarian/vegan diet and body composition. The 22 included studies, published from 2017 to 2021, included a total of 436,178 participants, which corresponds to a large sample size and wide geographic coverage. Several interventional and cross-section studies directly or indirectly assessed the effect of vegetarian/vegan diets on the body composition compared to omnivorous diets. The study by Jakse et al., involved an intervention with a low-fat vegetable-based diet and vegetable meal replacement (intervention group) and an omnivorous diet. These authors observed that the intervention group lost a significantly larger amount of weight, body fat, and visceral fat. Similar studies [13,14,15,16,17,18,20] choosing low-fat plant-based diets for the intervention group observed statistically significant reductions in body weight, BMI, and, in some cases, fat mass. These findings reinforce this potential association between low-fat diets and significant weight loss [33]. Other cross-sectional studies [5,25,26,27,28,29,30] also observed a significantly greater reduction in weight and BMI in individuals consuming plant-based diets. Karlsen et al. [34] found that the BMI reduction is more pronounced in individuals who followed this type of diet for more than one year. These results are in line with the EPIC-Oxford cohort study reporting that vegans and vegetarians lost weight after a 5-year follow-up trial. Lee et al. [12] performed a 10-day intensive health promotion study where individuals frequently performed physical activity and consumed a vegetarian or an omnivorous diet. At the end of the intervention, weight and BMI was found to be significantly reduced in the vegetarian diet group. However, psychological variables were not assessed, meaning that other factors beyond diet might contribute to the reported results. Recently Dinu et al. [19] compared the Mediterranean and vegetarian diets and suggested that statistically significant differences between these two regimens could not be detected in a first intervention. This study reported that after a second intervention, significant reductions in body weight, BMI, and fat mass were detected in both groups. Low caloric density and reduced levels of saturated fatty acids have been reported to explain these impacts of vegetarian/vegan diets on body weight and BMI [35]. The involvement of other nutrients such as higher mono- and polyunsaturated fat or fiber content has also been reported [35]. The lack of statistically significant differences between individuals consuming plant-based and omnivorous diets in body composition was found in four studies. The study by Sofi et al. [22] used a low-calorie diet intervention to reduce weight and cardiovascular risk. No statistically significant differences could be found for weight loss, BMI, and fat mass between the two groups. Shah et al. [23] found no differences in weight loss, BMI, and waist circumference between the vegan diet and the American Heart Association recommended diet in individuals with coronary artery disease. However, the American Heart Association recommended diet was created specifically for individuals with cardiovascular diseases [36], while the same does not apply to the vegan diet. The studies by Pinto et al. [31] and Heiss et al. [32] found no statistically significant differences regarding body composition between vegetable-based and omnivorous diets. However, it is not possible to establish a causal relationship with the results obtained due to the type of study conducted. One explanation for no statistical differences observed in different studies between vegetarian diets and body composition may be that they originate from developing countries where the diet is plant-based, or are those reliant on the Mediterranean diet for the obvious reason. However, in the present review, the five papers that did not find any statistically significant positive association all originate from developed countries. It is also important to understand that there is no clear consensus on the effects of plant-based diets on body composition. A recent study reported a relationship between being vegetarian/vegan and higher levels of fat mass and visceral adipose tissue, although these were non-statistically significant differences [37]. It is also noteworthy that all these studies presented various limitations. As an example, although the vast majority of studies have assessed body composition through dual-energy X-ray absorptiometry, some studies used self-reported measurements [25,27], normal scales [5,28], or the bioimpedance method [11,19,21,22,28,30,31]. Physical activity and lifestyle are not included as determinants in most of the studies. In these conditions, any comparisons involve significant bias. Other limitations are related to cross-sectional studies, which do not allow for establishing a causal relationship. In these same studies, there was a greater discrepancy in the sample (population) size, with some samples being very small [5,31] and others with significant heterogeneity between the dietary groups [25,26,30]. However, the fact that most of the studies included in this review were randomized clinical trials is a strong point in support of the scientific evidence presented.

## 5. Conclusions

Results gathered in this review suggest that vegetarian/vegan diets are associated with weight loss and BMI reduction and, in a few cases, with fat mass modifications. Our results also suggest that research on these matters should aim for larger and more representative numbers of vegetarians and vegans regarding the general population. It is also necessary to assess not only the effect of plant-based diets on body composition but the positive or negative contribution of each food group and nutrients within these same diets.

## Figures and Tables

**Table 1:** Summary of the experimental studies included in the present review.

Reference	Type of Study	Population/ Methods	Main Findings
Jakse et al., 2017 [24]	Nonrandomized Controlled Trial	325 participants: control group (*n* = 84) and intervention group (*n* = 241).	“The intervention group lost a significantly larger amount of weight, body fat, and visceral fat. The relative proportion of muscle mass and total body water increased relative to the control group.”
Lee et al., 2017 [12]	Randomized Controlled Trial	30 women: intervention group (vegetarian diet, *n* = 16) and control group (normal diet of participant, *n* = 14). Duration: 10 days	“Bodyweight and BMI decreased significantly in the intervention group compared with that in the control group.”
Wright et al., 2017 [13]	Randomized Controlled Trial	49 participants: intervention group (*n* = 25) and control group (*n* = 24). Duration: 6 months to 1 year	“At 6 months mean intervention BMI reduction was 4.4 kg/m^2^ and from 6 to 12 months intervention BMI increased non-significantly by 0.4 kg/m^2^. For weight, intervention reduction at 6 months was 12.1 kg, and at 12 months was 11.5 kg.”
Kahleova et al., 2018 [14]	Randomized Controlled Trial	75 participants: intervention group (*n* = 38) or a control group (*n* = 37). The intervention followed a low-fat vegan diet and the control group made no diet changes. Duration: 16 weeks.	“Parameters such as BMI, fat mass and especially visceral fat volume were significantly reduced in the intervention group.”
Kahleova et al., 2018 [15]	Randomized Controlled Trial	75 Participants: intervention group (plant-based high-carbohydrate, low-fat (vegan) diet; *n* = 38) or control group (current diet; *n* = 37). Duration: 16 weeks.	“In vegan group, there was a decrease in weight and in fat.”
Martínez-Rodríguez et al., 2018 [21]	Randomized Controlled Trial	21 women with fibromyalgia: Group A (core stabilization exercises + Lacto-vegetarian diet, *n* = 7); group B (stabilization exercises with ultrasound turned off and without the use of conductive gel + Lacto-vegetarian diet, *n* = 7) and group C (control, *n* = 7). Duration: 4 weeks.	“Group A, compared to groups B and C, showed significant changes, at the end of the intervention, in pain reduction and body composition. There was an increased muscle mass and decreasing fat mass.”
Sofi et al., 2018 [22]	Randomized Controlled Trial	118 participants: ovolactovegetarians group (*n* = 60) and Mediterranean diet group (*n* = 58). Duration: 2 periods of ≥3 months.	Weight loss was identical in both groups (vegetarians: −1.88 kg; Mediterranean diet: −1.77 kg). Similar results were observed for BMI and fat mass.
Kahleova et al., 2018 [14]	Randomized Controlled Trial	75 overweight participants: vegan diet (*n* = 38) or a control diet (*n* = 37). Duration: 16 weeks.	“Reductions in BMI, body weight, fat mass, and visceral fat volume in the vegan group.”
Shah et al., 2018 [23]	Randomized Controlled Trial	100 participants with angiographically: vegan diet (*n* = 50) or American Heart Association recommended diet (*n* = 50).	“No differences were observed in weight loss, BMI, and waist circumference reduction.”
Kahleova et al., 2019 [20]	Randomized Controlled Trial	75 overweight participants: low-fat vegan (*n* = 38) or control diet (*n* = 37). Duration: 16 weeks.	“A low-fat vegan diet was associated with decreased fat mass, insulin resistance, and enhanced insulin secretion.”
Kahleova et al., 2020 [17]	Randomized Controlled Trial	115 participants overweight: vegan group (*n* = 65) and control group (*n* = 50). Duration: 16 weeks	“Bodyweight decreased in the vegan group, mainly due to a reduction in fat mass and visceral fat.”
Kahleova et al., 2020 [18]	Randomized Clinical Trial	Overweight or obese participants: intervention group (*n* = 117), following a low-fat vegan diet, and control group (*n* = 106). Duration: 16 weeks.	“Mean body weight, fat mass and visceral fat volume decreased in the intervention group.”
Dinu et al., 2020 [19]	Randomized Controlled Trial	107 participants: vegetarian diet (*n* = 54), and Mediterranean diet (*n* = 53). Duration: 2 periods of ≥3 months.	“Reduction in body weight, BMI, and fat mass was observed in both groups, with no difference between the two diets.”

Abbreviations: body mass index (BMI), body fat percentage (%BF).

**Table 2:** Summary of the descriptive studies included in the present review.

Reference	Type of Study	Population/ Methods	Main Findings
Pinto et al., 2017 [31]	Comparative Study	23 participants following a vegan diet for ≥2 years compared with 24 omnivorous participants.	“No significant differences in mean BMI or other markers of body composition (%BF).”
Vanacore et al., 2018 [5]	Cross-sectional	30 participnats: vegetarians (*n* = 10); vegans (*n* = 10) and omnivores (*n* = 10).	“Decrease in muscle mass index and lean body mass between vegan vs omnivorous groups.”
Tong et al., 2018 [30]	Cross-sectional	Data from UK Biobank cohort participants. Caucasian individuals: 6 diet groups (regular meat-eaters, low meat-eaters, poultry eaters, fish eaters, vegetarians, and vegans). British Indian population: 2 diet groups (meat-eaters and vegetarians).	“White population who were poultry eaters, fish eaters, vegetarians, or vegans generally weighed less and had a lower BMI, waist and hip circumference and body fat percentage than the regular meat-eaters. British Indian vegetarian women had a slightly lower body weight and lower lean mass than meat-eaters.”
Jaceldo-Siegl et al., 2019 [25]	Cross-sectional	3475 Hispanic/Latino adults: vegan (*n* = 202), vegetarian (*n* = 664), pesco-vegetarian (*n* = 409), semi-vegetarian (*n* = 227) and nonvegetarian (*n* = 1973).	“Compared to the nonvegetarian, estimated BMI were lower among vegans, vegetarians, pesco-vegetarians, and semi-vegetarians. Plant-based diets were associated with lower BMI.”
Karlsen et al., 2020 [26]	Cross-sectional	8226 participants: whole food plant-based (*n* = 2141), vegan (*n* = 1584), Paleo (*n* = 1202), try to eat healthy (*n* = 968), vegetarian and pescatarian (*n* = 814), whole food (*n* = 690), Weston A. Price (*n* = 458) and low-carb (*n* = 369).	“BMI was lower among longer-term followers (≥1 year) of whole food, plant-based, vegan, whole food, and low-carb diets compared with shorter-term followers. Among those following their diet for 1–5 years, BMI were lower for all groups compared with try to eat healthy group.”
Matera, 2020 [27]	Cross-sectional	370 participants: 188 vegetarian and 182 omnivorous.	“Vegetarians had a lower BMI.”
Heiss et al., 2020 [32]	Cross-sectional	124 Participants: 72 omnivores, 27 meat-reducers, 20 vegetarians, and 5 vegans.	“Vegetarians reported lower BMI compared to meat-reducers.”
Saintila et al., 2020 [28]	Cross-sectional	149 participants: vegetarians (*n* = 62) and nonvegetarians (*n* = 87).	“Vegetarian males had a lower weight, waist circumference and BMI than nonvegetarian males.”
Jakse et al., 2021 [29]	Cross-sectional	Two groups: vegan (*n* = 51) and non-vegan (*n* = 29).	“All anthropometric and body composition variables were significantly lower in the vegans than non-vegans, except for body height.”

Abbreviations: body mass index (BMI), body fat percentage (%BF).

## Data Availability

Not applicable.
